# Correction: Nanoarchitectonics of p-type BiSbTe with improved figure of merit *via* introducing PbTe nanoparticles

**DOI:** 10.1039/d1ra90170b

**Published:** 2021-12-02

**Authors:** Yuanyue Li, Mengna Ren, Zhongsen Sun, Zhao Yao

**Affiliations:** College of Electronic and Information, Qingdao University Qingdao 266071 China sunzs@qdu.edu.cn yao9074@hotmail.com

## Abstract

Correction for ‘Nanoarchitectonics of p-type BiSbTe with improved figure of merit *via* introducing PbTe nanoparticles’ by Yuanyue Li *et al.*, *RSC Adv.*, 2021, **11**, 36636–36643, DOI: 10.1039/D1RA07138F.

The authors regret that affiliation *a* was incorrectly given in the original article. The correct affiliation is shown here.

In the sentence beginning “For instance, PF of the sample with *χ* = 0.5 wt% is 33.8…” incorrect units were given. The corrected sentence is as follows: For instance, PF of the sample with *χ* = 0.5 wt% is 33.8 μW cm^−1^ K^−2^ at 482 K, which is ∼33% larger than that of the BST matrix (25.5 μW cm^−1^ K^−2^).

In the sentence beginning “For instance, as to the sample with *χ* = 0.5 wt%, *κ* decreases from 1.10…” incorrect units were given. The corrected sentence is as follows: For instance, as to the sample with *χ* = 0.5 wt%, *κ* decreases from 1.10 to 0.99 W m^−1^ K^−1^ at 482 K (a ∼10% decline).

The Royal Society of Chemistry apologises for these errors and any consequent inconvenience to authors and readers.

## Supplementary Material

